# Severe tracheobronchomalacia misdiagnosed as an asthma exacerbation

**DOI:** 10.1093/omcr/omad032

**Published:** 2023-04-20

**Authors:** Hossny Alaws, Rami Arfoosh

**Affiliations:** Department of Internal Medicine, Northeast Georgia Medical Center, Gainesville, GA 30501, USA; Department of Medicine, Augusta University/University of Georgia Medical Partnership, Augusta, GA 30912, USA

A 61-year-old female presented to the Emergency department with a cough and shortness of breath. She has a medical history of asthma with recurrent asthma exacerbations and prior intubation. At the time of evaluation, she was tachycardic and tachypnic. Physical examination revealed upper airway wheezing worse when laying down. Plain chest radiographs were unremarkable. She was diagnosed with asthma exacerbation and was admitted for observation. She was started on a course of antibiotics, steroids and bronchodilators.

**Figure 1:**
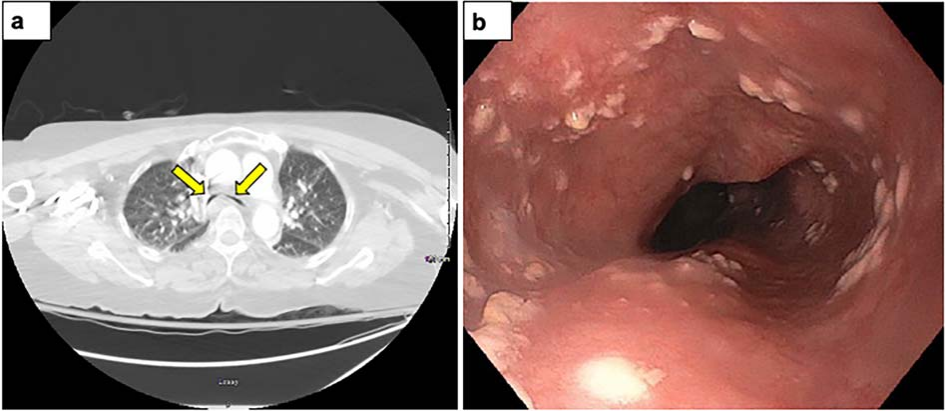
(**a**) Transverse computed tomography image showing severe tracheobronchomalacia with collapsing right and left main bronchi (arrows). (**b**) Bronchoscopic image of the trachea with decreased anteroposterior diameter showing evidence of tracheobronchomalacia.

Her symptoms did not improve despite treatment. Given her tachycardia and tachypnea, a computed tomography angiography was obtained to rule out a pulmonary embolus, and showed a new finding of severe tracheomalacia with greater than 90% narrowing of the trachea (Fig. 1a and b). Her symptoms improved with continuous positive airway pressure. She was later discharged home with nightly continuous positive airway pressure (CPAP) as a bridging therapy while awaiting airway stenting. She later underwent bronchoscopic evaluation which showed tracheal collapse with 80% airway occlusion during coughing indicative of tracheobronchomalacia.

Tracheobronchomalacia is characterized by excessive collapsibility of the trachea and bronchi particularly during exhalation [1]. Causes include congenital, recurrent intubation, indwelling tracheostomy, respiratory infections and extrinsic tracheal compression [1, 2]. These insults lead to weakening of the cartilage supporting the trachea leading to collapsibility [3]. Patients report shortness of breath, cough with deep inspiration, episodic

choking and recurrent pulmonary infections. Inspiratory wheezing or stridor, usually with deep inspiration or expiration may be the only notable physical exam finding [1, 3, 4]. Additional findings include early cessation of expiratory flow during forced expiration [3]. History and physical examination are nonspecific. Dynamic computed tomography (CT) and flexible bronchoscopy are the mainstays of diagnosis [1]. Tracheobronchomalacia (TBM) can be confused with or exacerbated by respiratory infections or obstructive lung conditions such as asthma [1]. Therefore, recognizing this condition can prevent the overtreatment of asthma exacerbations.
